# Ruptured AAA: bridging the gap between international guidelines and local clinical realities

**DOI:** 10.1007/s00423-024-03441-6

**Published:** 2024-08-20

**Authors:** Summer Hassan, Taylor Frost, Russell Bourchier

**Affiliations:** 1 The Department of Vascular Surgery, Auckland City Hospital, 2 Park Road, Auckland, New Zealand; 2 University of Auckland, Auckland, New Zealand

**Keywords:** Abdominal aortic aneurysm, Ruptured AAA, AAA size at rupture, Mortality outcomes, AAA rupture risk

## Abstract

**Background:**

Treatment of asymptomatic Abdominal Aortic Aneurysms (AAA) presents a clinical challenge, requiring a delicate balance between rupture risk, patient comorbidities, and intervention-related complications. International guidelines recommend intervention for specific AAA size thresholds, but these are based on historical trials with limited female representation. We aimed to analyse disease characteristics, AAA size at rupture, and intervention outcomes in patients with ruptured AAA from 2009 to 2023 to investigate the gap between guidelines and local realities.

**Methods:**

This single-centre retrospective cohort study analysed electronic health records of patients treated for a ruptured AAA, excluding those who were managed palliatively. The study assessed patients’ demographics, risk factors, comorbidities, clinical presentation, radiological characteristics, and outcomes.

**Results:**

Of 164 patients (41 females, 123 males, median age 73.5), 93.3% presented with abdominal or back pain. The median AAA size at rupture was 8.0 cm in males and 7.6 cm in females. No significant correlations were found between demographic characteristics, risk factors, AAA size, repair modality, and outcomes. Trends show a decline in AAA prevalence and rupture rates, aligning with global health initiatives. Post-intervention survival rates at 30 days were 70.7% (67.5% in males and 80.0% in females), and at 2 years were 65.85% (61.7% in males and 70.0% in females).

**Conclusion:**

Evolving AAA trends and improved post-intervention survival rates warrant a critical reassessment of existing intervention recommendations. Adjusting intervention thresholds to larger sizes may be justified to optimise the risk-benefit ratio.

## Introduction

Treatment of asymptomatic Abdominal Aortic Aneurysms (AAA) often poses a complex clinical dilemma, necessitating a nuanced balance between the risk of rupture, patients’ underlying comorbidities, and the potential intervention complications. The decision to intervene electively on AAA becomes more intricate in light of evidence asserting that long-term survival benefits, even after treatment, remain lower in patients with electively repaired AAA compared to their matched general populations without AAA [1–3]. The European Society for Vascular Surgery (ESVS) and the American Society for Vascular Surgery (SVS) recommend elective intervention when AAA reaches a threshold of 5.5 cm in men and 5.0 cm in women [4, 5]. Four randomised clinical trials were the cornerstone evidence for the development of these guidelines: two large multicentred randomised controlled trials of early open elective surgery versus surveillance, the UK Small Aneurysm Trial (UKSAT) and the American Aneurysm Detection And Management study (ADAM), and two smaller trials of endovascular repair versus surveillance; the Comparison of surveillance vs. Aortic Endografting for Small Aneurysm Repair (CAESAR) Trial, and the Positive Impact of endoVascular Options for Treating aneurysm early (PIVOTAL) study [6–9]. It is pertinent to acknowledge that the inclusion of a limited number of females in these trials has led to a reliance on comparatively weaker evidence for recommendations regarding female patients. The UKSAT reported an aneurysmal rupture rate of 1% per year in their cohort of patients with small aneurysms of 4.0–5.5 cm undergoing surveillance. It contrasts with a perioperative mortality of 5.6% in the immediate open repair cohort. Similarly, the ADAM study revealed a perioperative mortality of 2.7% in the open repair cohort compared to the rupture rate of 0.6% per year in the surveillance group for small AAAs (4.0 cm to 5.5 cm). These results affirm that immediate repair confers no advantage for small AAAs, irrespective of the chosen modality.

The current indication for intervention in AAAs larger than 5.5 cm is based on historical findings of alarming mortality rates associated with rupture —up to 80% in women and 70% in men [10]. ESVS 2011 guidelines indicated the yearly rupture risk escalating from 1% in AAA < 5.0 cm to 33% in aneurysms > 7.0 cm [11]. A recent meta-analysis scrutinising rupture rates based on size across 1514 patients from 11 studies indicates a significant departure from previous reports. Parkinson et al. found yearly rupture rates to be 3.5% in AAAs 5.5 to 6.0 cm, 4.1% in AAAs 6.1 to 7.0 cm, and 6.3% in AAAs > 7.0 cm [12]. Interestingly, the recently released ESVS 2024 guidelines acknowledge that adjusting this threshold to 6.0 cm may prove safe in the future as more evidence emerges. Their committee has therefore issued a strong negative recommendation for intervening electively on AAA < 5.5 cm and downgraded the recommendations for intervention at the 5.5 cm threshold to be a consideration of repair in men (from Class I to Class IIa) as the randomised clinical trials underlying this recommendation only showed that intervening on AAA < 5.5 cm is of no added benefit [13].

The lower rupture rates may be attributed to advancements in mitigating risk factors known to precipitate AAA rupture, including the decline in smoking prevalence and improved recognition and management of cardiovascular diseases [14, 15]. Furthermore, AAA screening programs implemented in various countries facilitate timely intervention and prevent AAA-related mortality. For instance, The 10-year follow-up results from the United Kingdom Multicentre Aneurysm Screening Study (MASS) revealed that screening men aged 65–74 for AAA led to a 48% relative risk reduction in deaths related to AAA over 10 years. The study demonstrated that the benefit of screening was maintained over time, with the mortality reduction seen in earlier years persisting in later years [16]. Similarly, in Denmark, a study focusing on asymptomatic men aged 64–73 years confirmed the favourable outcomes observed in the UK trials. The study demonstrated that screening for AAA resulted in a 66% reduction in AAA-related mortality at 14 years of follow-up [17, 18]. The systematic review and meta-analysis by Ying et al. consolidated evidence from randomised trials and cost-effectiveness analyses [19]. The review included five studies with a total of 175,085 participants aged 64 to 83 years, with a mean follow-up of 10.6 years. The results indicated that AAA screening led to a reduction in all-cause mortality, AAA-related mortality, and emergent AAA repair. The number needed to screen to prevent 1 AAA-related death per 10 years ranged from 209 to 769 individuals [19].

Considering these evolving trends in AAA prevalence, diagnosis and management, our retrospective single-centre cohort study aims to investigate the disease characteristics and AAA size at rupture for patients presenting to our regional vascular service at Auckland City Hospital from 2009 to 2023. The study further seeks to correlate these characteristics with patient outcomes, contributing valuable insights to the ongoing discourse on AAA management.

## Methods

### Study design

This retrospective cohort study investigates the demographic and disease characteristics of patients presenting with ruptured AAA between 2009 and 2023 at Auckland City Hospital. Patients were included from 2009 onwards to improve the accuracy of AAA measurements, as radiological modalities were better utilised during this period. Additionally, the study seeks to correlate these characteristics with patient outcomes. The study utilised electronic health records of patients diagnosed with ruptured AAA during this timeframe.

### Study setting

The study was conducted at Auckland City Hospital, a single-centre tertiary care facility serving as one of at least 5 regional vascular surgery referral centres and equipped with advanced diagnostic and therapeutic facilities, including a hybrid theatre, for comprehensive management of vascular emergencies.

### Data collection

Patients included in the study are 18 years or older, diagnosed with ruptured AAA between 2009 and 2023, and have complete records. Importantly, only patients who were deemed appropriate for surgical or endovascular intervention and subsequently treated were included. Patients managed palliatively without intervention were not included in this study. Patients were excluded from the study if they had definite evidence of mycotic rupture of AAA or non-ruptured AAA or if they presented with other aortic pathologies. Notably, two patients with rAAA and a diameter < 55 mm were included due to the lack of definitive evidence of mycotic aneurysm, despite the operating surgeon’s concern for mycotic features. Intra-operatively collected samples and repeated blood cultures during their inpatient stay showed no microbial growth, justifying their inclusion in this study. The following variables were collected: Demographics, including ethnicity, risk factors and comorbidities, clinical presentation, and radiological characteristics of AAA. AAA size at rupture as measured by ultrasound, CTA, or intra-operatively. Interventional data and peri-interventional complications. Mortality at 30 days, 90 days, and two years. Intensive care and inpatient hospital stay duration, and post-operative complications.

### Limitations

Limitations of the study include the retrospective design, reliance on electronic health records, and the single-centre nature, which may impact the generalizability of findings. Furthermore, certain specific data points were not comprehensively collected due to limitations in the patient records, such as the intraoperative vasopressor support and the time from diagnosis to intervention. In addition, the size of AAA at rupture was obtained through variable modalities.

## Statistical analysis

### Descriptive statistics

These provided a comprehensive overview of the demographic and clinical characteristics of patients presenting with ruptured AAA from 2009 to 2023. This included measures of central tendency and dispersion for continuous variables and frequency distributions for categorical variables. Results were expressed as a median ± standard deviation (SD).

### Correlation analysis

Statistical correlations were explored between patients’ sex and the size of AAA at rupture. Additionally, the relationship between sex and mortality outcomes was investigated. Similar correlation analyses were conducted with ethnicity as a variable to understand potential disparities in AAA size at rupture and outcomes among different ethnic groups.

### Grouping analysis

Patients were grouped based on AAA size at rupture (< 5.5 cm, 5.6–6 cm, 6.1–7.0 cm, > 7.0 cm) and analysis to assess each group’s distribution and percentage representation. This grouping allowed for the exploration of the impact of AAA size on patient outcomes. In addition, maximum AAA diameter density plots were constructed to visually depict the distribution of AAA sizes at rupture within the cohort. Age density plots were generated to illustrate the age distribution of the patients. Furthermore, Kaplan-Meier estimates of survival were calculated to compare the survival rates of patients with ruptured AAA who survived at 30 days post AAA repair to those of a matched sex and year of birth in New Zealand. The New Zealand population for this comparative analysis was defined using national demographic data from Statistics New Zealand (Stats NZ), which includes age, sex, and ethnicity-specific mortality rates. This approach allowed for a meaningful comparison between the survival rates of our cohort and the general New Zealand population, accounting for key demographic factors [20]. This analysis enabled crude assessment of long-term survival trends following intervention.

## Results

### Descriptive statistics

**Fig. 1:**
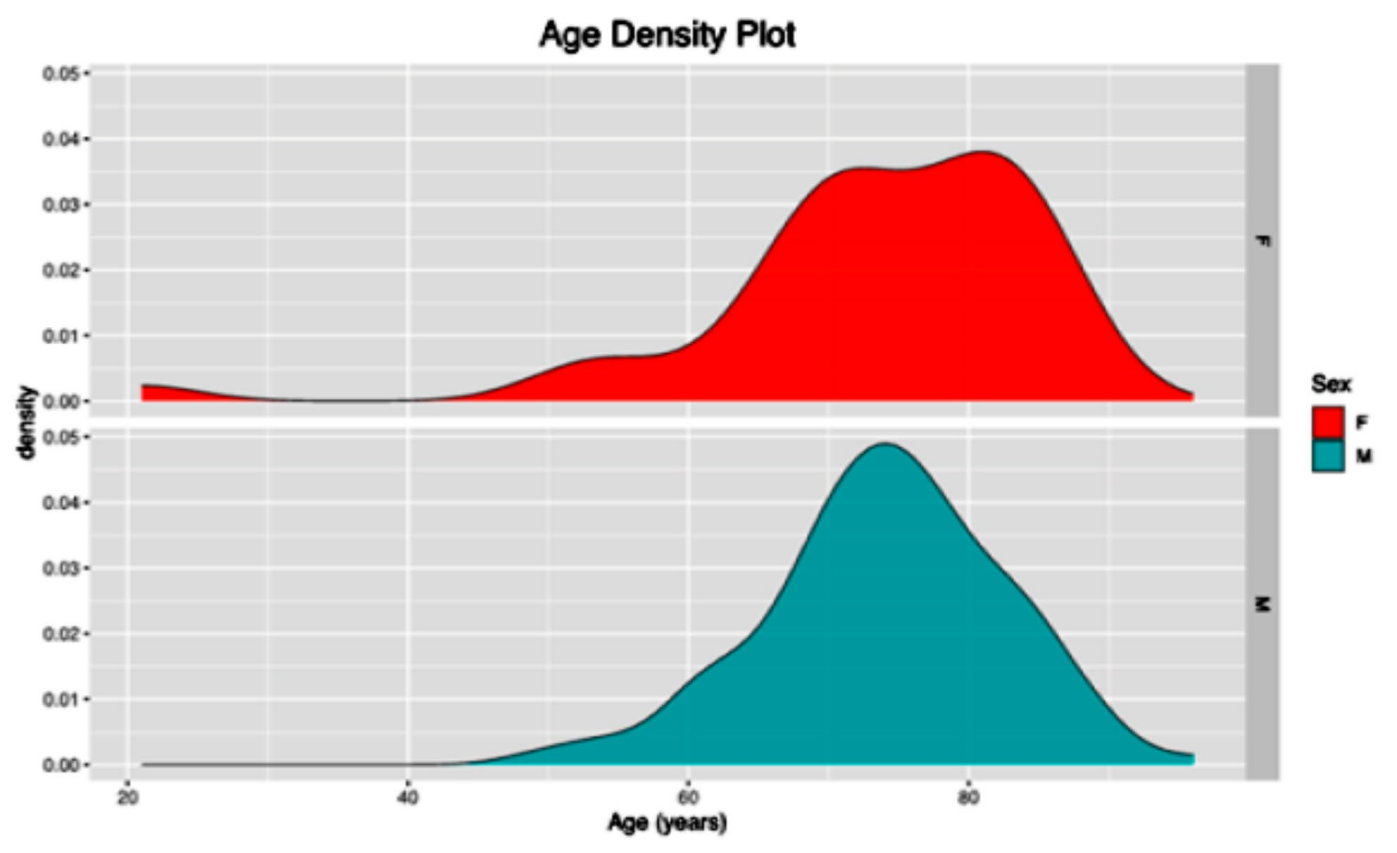
Age density plot per sex

**Fig. 2:**
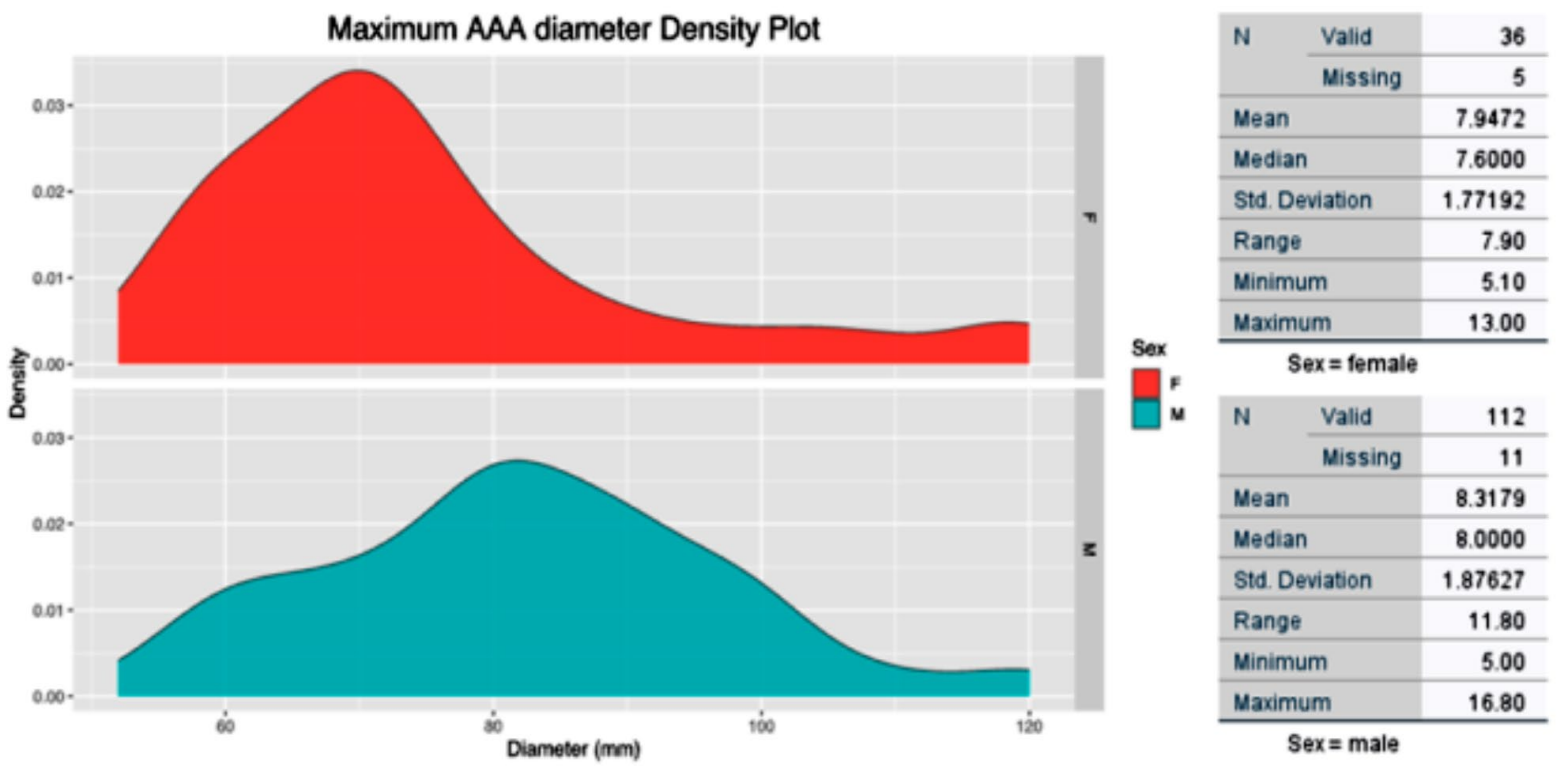
Descriptive statistics and density plot for maximum AAA size at rupture grouped by sex

In this study, 164 patients (41 females [25.0%] and 123 males [74.0%]) were enrolled, with a median age of 73.5 ± 16.53 (shown in Fig. 1). The ethnic distribution included 122 (74.4%) of European descent, 28 (17.1%) Māori, 11 (6.7%) Pacifica, and 3 (1.8%) of Asian ethnicity. Assessment of risk factors and comorbidities revealed that 53.0% had a history of regular smoking, 67.1% were hypertensive, 12.8% were diabetics, and 49.4% had a cardiac comorbidity. Unsurprisingly, most patients presented with abdominal or back pain (93.3%), 63.4% were hemodynamically unstable, defined as the presence of systolic blood pressure less than 90 mmHg, a heart rate exceeding 120 beats per minute, requirement for inotropic or vasopressor support, and clinical signs of shock such as altered mental status, oliguria, or cool extremities [21], and 5.5% suffered from end-organ dysfunction at presentation. The median size of AAA at rupture was 8.0 ± 1.87 cm in males and 7.6 ± 1.77 in females. The density plot for AAA maximum size at rupture is shown in shown in Fig. 2.

Patients were grouped based on the maximum diameter size at rupture: 3 patients (1.82%) had AAA < 5.5 cm, 17 patients (10.36%) had AAA sized 5.6–6.0 cm, 26 patients (15.85%) had AAA sized 6.1–7.0 cm, 103 (62.80%) had AAA of > 7.0 cm, and 15 (9.14%) had missing AAA size data on electronic records. CTA measured AAA sizes in 64.0% of patients, USS in 28.7%, and intra-operatively in 5.5% of patients. Notably, 18 patients (10.97%) experienced an interval rupture during surveillance, with 5 awaiting custom EVAR, 5 lost to follow-up, 3 declined elective intervention, 3 fell below the threshold for intervention, and 2 deemed unfit for intervention. The mean AAA diameters for patients experiencing interval rupture were 7.1 cm for males (*n* = 12) and 6.7 cm for females (*n* = 6).

Open surgical repair (OSR) was utilised in 136 (82.9%) patients, compared to 17 (10.4%) urgent Endovascular Aortic Repair (EVAR). The study found that the average use of EVAR per year for ruptured AAA (rEVAR) remained stable throughout the study duration, with an average of 1.2 rEVAR procedures performed per year. Eleven patients (6.7%) who proceeded for open repair arrested with failed resuscitation efforts “mors in tabula”.

Intra-operative details showed that proximal clamping was at the supra-coeliac level in 30.5% of patients, suprarenal in 12.8%, juxtarenal in 12.1%, and infrarenal in 44.7% of the patients. The median clamp duration was 22.5 ± 29.15 min. Patients who received OSR had proximal anastomosis at the supra-coeliac aorta (4.0%), juxtarenal (15.2%), or infrarenal aorta in 80.8% of the cases. The distal anastomosis sites were at the aortic bifurcation (56.6%), distal aorta (17.5%), common iliac artery (18.2%), internal iliac artery (0.7%), external iliac artery (4.2%), common femoral artery (12.3%), and the superficial femoral artery (0.7%). The median estimated blood loss was 3.1 ± 4.27 L, and the median operating duration was 170.0 ± 83.78 min.

**Fig. 3:**
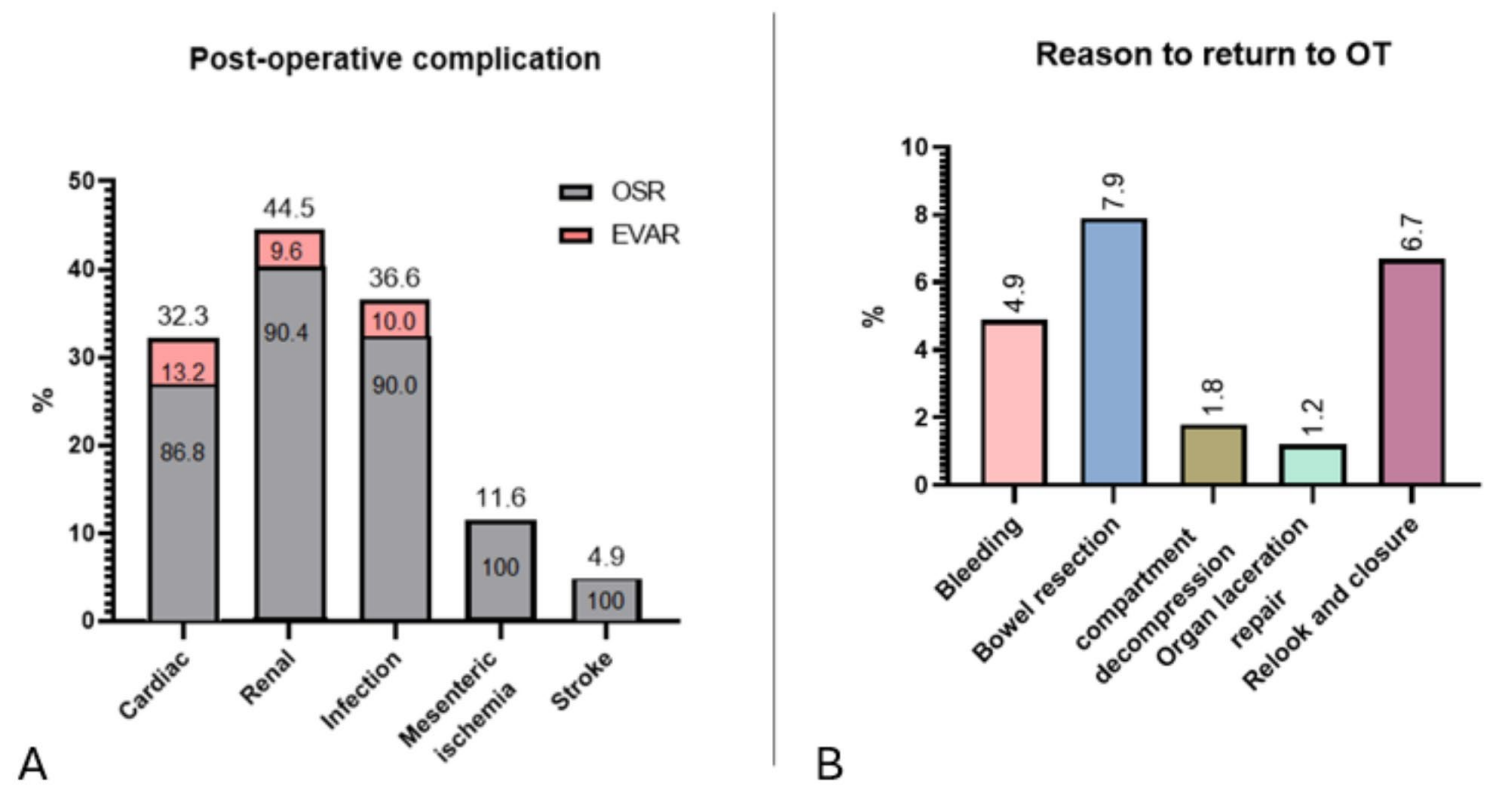
Summary of reasons for return to theatre and post-operative complications

Intra-operative complications occurred in 45 (27.4%) patients, manifesting as intra-operative cardiac arrest (8.5%), organ laceration (7.9%), torrential bleeding as described by the operator (7.3%), and intra-operative death (3.7%). In the study cohort, 44 (26.8%) returned to theatre; all patients who required a re-intervention were from the OSR group, with the exception of one patient in the EVAR group returning to theatre to control intra-abdominal bleeding. Return to theatre for abdominal closure was required in (6.7%) of patients, while (4.9%) returned to theatre to control bleeding. Furthermore, (7.9%) returned to theatre for bowel resection due to bowel ischemia. Post-operative complications were most pronounced in the OSR group compared to the EVAR group. These included cardiac events in (32.3%) of patients, while (44.5%) experienced a renal injury post-operatively, (36.6%) had a confirmed infective complication, (11.6%) developed mesenteric ischemia, and (4.9%) developed a stroke post-operatively. The percentage of these complications in the OSR and EVAR group, as well as the causes for return to theatre, is demonstrated in Fig. 3.

**Fig. 4:**
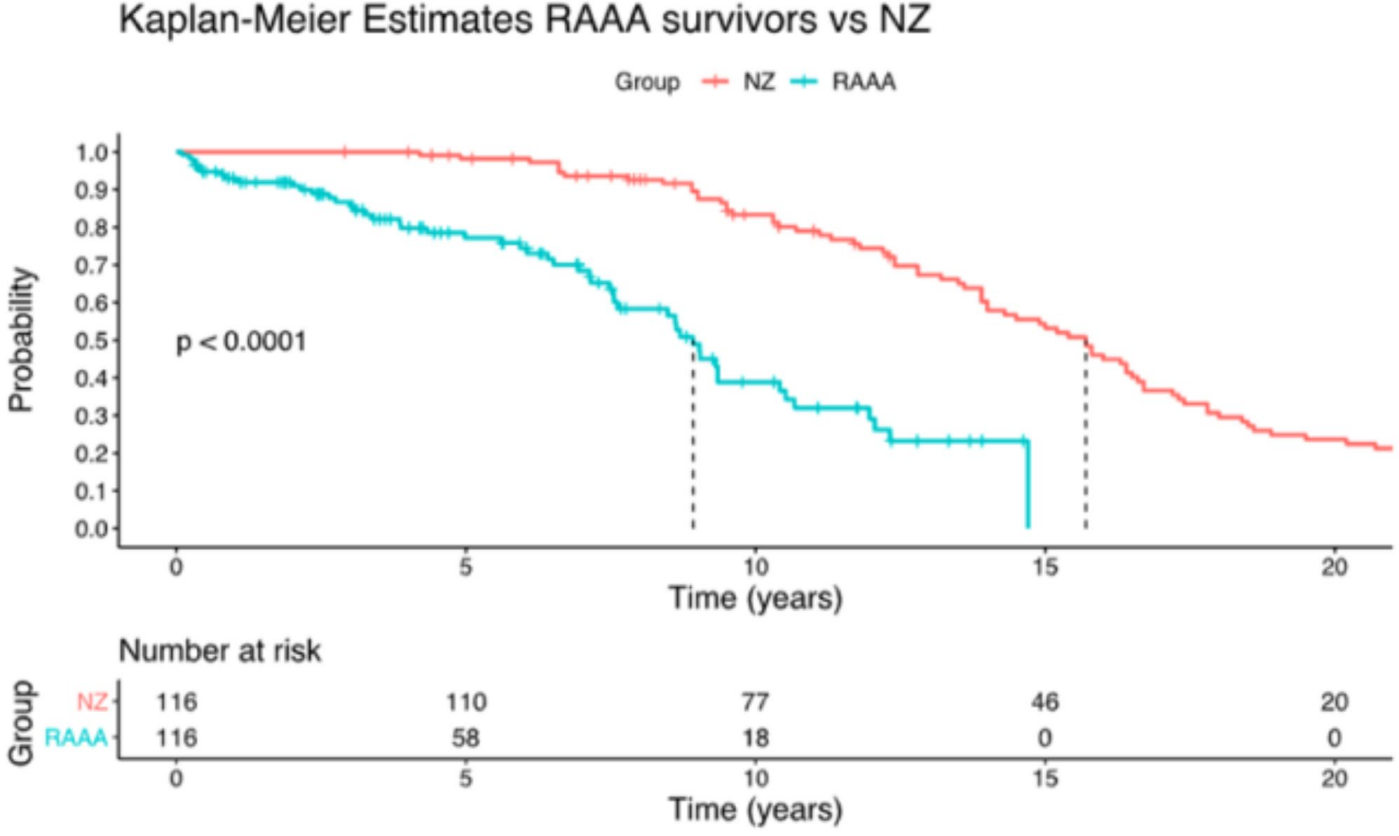
Kaplan-Meier and survival data in ruptured AAA cohort compared to matched New Zealand (NZ) population

Overall, the 30-day survival rate was 70.7% (67.5% in males and 80.0% in females). In the OSR group, 75.0% and in the EVAR group, 88.2% survived 30 days post-operatively. No change in survival was observed at 90 days. The 2-year survival rate was 65.85% (61.7% in males and 70.0% in females), with 2.43% of patients lost to follow-up. Kaplan Meier survival estimates comparing patients who survived more than 30 days after intervention (*n* = 116) to the matched New Zealand population (shown in Fig. 4) showed a median survival post AAA rupture of 8.9 years compared to predicted NZ mean survival of 15.7 years (*p* < 0.001; Hazard ratio 4.38 +/- 0.23).

The median duration of intensive care stay was 3.0 ± 7.12 days, and the median inpatient duration was 11.5 ± 14.16 days. Discharge destinations included home (40.9%), transfer to another hospital (20.1%), a rehab facility (6.7%), and rest home discharge (1.8%). No significant trends in 30-day or 2-year survival rates, post-operative complications, duration of inpatient and intensive care stay, or discharge destinations were observed over the study period from 2009 to 2023.

### Correlations among variables of interest

No statistically significant correlations were identified between patients’ sex or ethnicity and AAA size at rupture or mortality outcomes. Furthermore, no correlations were observed between risk factors such as smoking and hypertension and size at rupture or patient outcomes. In our patient population, neither the size of AAA at presentation nor the modality of repair yielded a statistically significant correlation with outcomes.

## Discussion

### Trends in AAA Prevalence and rupture Rates

The observed decline in AAA prevalence aligns with global efforts to improve health literacy and address key risk factors, particularly smoking [22]. Historical rates exceeding 5% are now substantially reduced to 1–2% and 1% in smoking females > 70 years old. This trend indicates the success of public health campaigns to mitigate risk factors associated with AAA [23–25]. As prevalence rates decline, a consequential decrease in rupture rates results in a significant shift from previous data. In the 1970s, rupture rates were reported to be 40% for AAAs > 6.0 cm, which decreased to 9.4% in the 1990s for AAAs 6.0 to 6.9 cm, and further reduced to 4.1% in AAAs 6.1 to 7.0 cm in recent studies [12, 26, 27].

### Survival rates post rupture repair

Interventions for ruptured AAA have shown a gradual yet positive trend, with a noteworthy decrease in mortality rates observed over time. In our study, the 30-day survival rates were 75.0% in OSR and 88.2% in the EVAR group, while the overall 2-year survival rates were 65.9%. These outcomes align with recent studies reporting a 5-year survival of 68.8–71.0% in patients undergoing EVAR for ruptured AAA [28, 29]. In a recent review including data from IMPROVE and AJAX trials, 30-day survival rates were 64.6% for EVAR and 62.6% for OSR in IMPROVE. At one year, EVAR had a survival rate of 58.9%, while OSR was 54.9%. For AJAX, 30-day survival rates were 79% for EVAR and 75% for OSR. However, at six months, survival rates dropped to 72% for EVAR and 69.5% for OSR [30]. These results resonate with our study findings, indicating a modest yet notable improvement in survival outcomes compared to a previous literature review from 1991 to 2006, which reported 51% perioperative survival [31, 32]. The improved mortality rates and the recognised positive outcomes in high-volume centres indicate that ongoing regionalisation efforts in vascular surgery may lead to even better outcomes for these patients.

In this study, the higher ratio of OSR relative to rEVAR is in keeping with data from other centres in New Zealand. This trend can be attributed to several factors. Firstly, a significant proportion of our patients (63.4%) presented with hemodynamic instability, precluding many from undergoing the CTA necessary for EVAR planning. The urgent nature of their condition often necessitated immediate surgical intervention, favouring OSR over EVAR. Additionally, patient preference influenced the choice of OSR, although this aspect was not specifically documented in our study. During the earlier years of our study duration, the option for EVAR was limited due to infrastructure and the availability of trained personnel. While the introduction of emergency rEVAR in New Zealand presents an appealing alternative to open repair for rAAA, the adoption has been slow and gradual due to limited resources, patient load and endovascular experience [33]. The study found that the average use of EVAR per year for ruptured AAA remains stable, with an average of 1.2 rEVAR procedures performed per year. This stable trend indicates that despite the benefits of EVAR, it has not significantly increased over time in our setting. Globally, EVAR has shown increasing utilisation for rAAA. A review of nine countries reported an average annual rate increase of rEVAR from 5.3 to 15.1% between 2005 and 2009 [34], with current annual rEVAR rates of 35–70% within EVAR-capable centres in Sweden and the US [35, 36]. Local studies, such as the one by Peek et al. [37], highlighted that ruptured AAA repairs are more expensive than elective repairs, adding another layer of complexity to the decision-making process. The higher costs and logistical challenges associated with emergency EVAR might have contributed to the continued preference for OSR in acute settings. In summary, the combination of patient factors, historical limitations in infrastructure and expertise, and local economic considerations have collectively contributed to the higher ratio of OSR relative to rEVAR in New Zealand.

### Considerations for intervention thresholds

The ongoing success in reducing AAA-related mortality prompts a critical re-evaluation of current international guidelines recommending intervention at a 5.5 cm or lower threshold. The latest ESVS guidelines recognise the changes in AAA trends and intervention outcomes, resulting in amending the wording from “recommending” elective intervention on AAA of 5.5 cm to “considering” intervention on AAA at this threshold [13]. Our data, reflecting a median size at rupture of 8 cm, suggests that the current guidelines may lead to an over-treatment of AAA. This is particularly relevant when considering the competing mortality risks of about 5% in elective repairs [38, 39]. Furthermore, this study highlights several factors that underscore the need for revisiting intervention thresholds. Firstly, despite international recommendations advising consideration of elective intervention at AAA thresholds of 5.5 cm, only 1.82% of our study population had a ruptured AAA at this size, with underlying aortic infection being a possible precipitating factor in 2 of the 3 patients. However, no definitive microbiological evidence could be elicited. Secondly, patient factors, including functional status and comorbidities, must be meticulously considered in determining the appropriateness of elective intervention. It is essential to recognise that patients with small AAAs may undergo a significant physiological insult during repair, potentially outweighing the benefits of intervening. Finally, our findings align with existing evidence indicating lower survival rates for patients with AAA, even after repair, compared to their matched populations. In our cohort, a notable proportion of patients presented with substantial comorbidities, including smoking diabetes, and importantly, 49% of our patient population had cardiac comorbidities. These comorbidities likely contributed to the observed poor long-term mortality rates, reflecting the overall health status and risk profiles of these patients rather than the outcomes of AAA repair alone. This study did not specifically match the patients’ comorbidities to the general population. However, the prevalence of cardiac comorbidities in patients over 60 years old is well-recognised [40]. Therefore, while comparing the long-term survival of patients with repaired rAAA to a demographics-matched population provides valuable insights, the potential bias introduced by the higher prevalence of comorbidities in the rAAA group must be considered and warrants future dedicated studies.

The study underscores the complexity of guiding AAA treatment solely based on aneurysm size and emphasises the importance of considering broader patient factors in decision-making. The observed trends in AAA prevalence, rupture rates, and post-intervention mortality outcomes suggest a potential need for revising current guidelines, prompting consideration for adjusting the elective intervention threshold to a larger size [41, 42]. It is important to note that our study is an observational and hypothesis-generating rather than a conclusive study. The findings should be interpreted within the context of a single-centre retrospective cohort study, and larger, multicentre studies are necessary to validate these observations and support any changes in clinical practice. Our discussion aims to highlight emerging trends and suggest a re-evaluation of current guidelines in light of these findings, not to recommend new thresholds definitively.

In conclusion, the dynamic nature of AAA disease trends and improved peri-intervention survival with lagging long-term survival benefits challenges existing paradigms. A careful reassessment of intervention thresholds is warranted.

## Data Availability

No datasets were generated or analysed during the current study.
